# Multi-Radio Based Rendezvous Technique for Heterogeneous Cognitive Radio Sensor Network

**DOI:** 10.3390/s21092997

**Published:** 2021-04-24

**Authors:** Md. Tahidul Islam, Sithamparanathan Kandeepan, Robin. J. Evans

**Affiliations:** 1 Department of Electronic and Telecommunications Engineering, RMIT University, Melbourne, VIC 3000, Australia; kandeepan@ieee.org; 2 School of Electrical and Electronic Engineering, The University of Melbourne, Melbourne, VIC 3010, Australia; robinje@unimelb.edu.au

**Keywords:** cognitive radio sensor network, multi-radio rendezvous, prime number theory, rendezvous probability

## Abstract

In a distributed cognitive radio (CR) sensor network, transmission and reception on vacant channels require cognitive radio nodes to achieve rendezvous. Because of the lack of adequate assistance from the network environment, such as the central controller and other nodes, assisted rendezvous for distributed CR is inefficient in a dynamic network. As a result, non-assisted blind rendezvous, which is unaware of its counterpart node, has recently led to a lot of interest in the research arena. In this paper, we study a channel rendezvous method based on prime number theory and propose a new multi-radio-based technique for non-assisted rendezvous with the blind and heterogeneous condition. The required time and the optimal number of radios for the guaranteed rendezvous are calculated using probability-based measurement. Analytical expressions for probabilistic guaranteed rendezvous conditions are derived and verified by Monte Carlo simulation. In addition, the maximum time to rendezvous (MTTR) is derived in closed form using statistical and probabilistic analysis. Under different channel conditions, our proposed solution leads to a substantial time reduction for guaranteed rendezvous. For the sake of over-performance of our proposed system, the simulation outcome is compared to a recently proposed heterogeneous and blind rendezvous method. The Matlab simulation results show that our proposed system’s MTTR gains range from 11% to over 95% for various parametric values of the system model.

## 1. Introduction

In just a few years, there has been growing interest in the potential future increase in the number of devices using next-generation (5G) communications [1]. The objective of integrating a large number of wireless sensor nodes is to be a part of the Internet of Things (IoT) evolution in the future [2,3]. A huge proportion of spectrum demand is anticipated for the smooth functioning of millions of wireless sensor devices in any geographical domain for the future generation of communication technologies. Traditional approaches to eliminating spectrum scarcity, such as enhanced bandwidth allocation, MIMO, full-duplex connectivity, appropriate cell strategy and so on [4]. However, the aforementioned methods are mostly dependent on resource optimisation, and the cost-efficiency of the parameters listed is still insignificant. The CR prototype, on the other hand, holds the promise of efficient dynamic exploitation of the underutilized spectrum [5]. In a cognitive radio sensor network (CRSN), cognitive functionality is integrated with sensor nodes to form a new sensor networking paradigm. Therefore, allowing unlicensed sensor nodes to broadcast in the licensed band was proposed as a way to increase spectrum efficiency and accommodate users in the unlicensed spectrum band [6]. A cognitive radio user (CRU) detects one or more empty channels to avoid interference in the first stage prior to CR communication. After that, fast and efficient blind identification of vacant common channels, which will support both transmission and reception, is one of the major challenges for any CRSN system. One of the basic processes of initiating data transfer in the CR phase is rendezvous, which ensures one universal channel between two communication devices. In the current literature, two rendezvous methods are popular—assisted rendezvous, where the nodes acquire cooperation from another node or a central controller, and non-assisted rendezvous, where there is no assistance from the network environment between nodes or devices. In the existing literature, two approaches for assisted rendezvous are identified—rendezvous based on a central controller [7,8] and cooperative rendezvous without central controller [9,10,11]. The former assisted rendezvous methods are performed merely based on the central controller node and without any assistance from other nodes in the network. On the other hand, assistance from other nodes is necessary for rendezvous in the latter case. The presence of a global common control channel (CCC) is required for all of the assisted rendezvous techniques described above. The prospect of achieving rendezvous with the help of CCC and nodes (central or general) appears to be very optimistic, mitigating all uncertainties. For reaching consensus on any common channel, the aforementioned assisted rendezvous approaches were primarily noticeable for their simplicity. However, several practical obstacles for assisted rendezvous are underlined [8,10,11], as follows—(i) lack of availability of CCC in a dynamically varying network environment; (ii) single point failure of a centralized controller and CCC; (iii) lack of scalability, flexibility and robustness of central controller; (iv) security problems for the central controller and CCC; (iv) inability of certain nodes to cooperate and (v) impracticality of having a central point for some physical spaces, such as a battlefield. As a result, the lack of the existence of assisted rendezvous leads us to focus on unassisted rendezvous as a real-life consequence.

Non-assisted rendezvous, on the other hand, is one that operates without the assistance of a central controller (or another node) and does not have a CCC. There are many approaches for non-assisted rendezvous techniques presented in the conventional references [12,13,14,15,16,17,18,19,20,21,22,23,24,25,26,27]. The general terminologies used for our articles related to rendezvous are summarized as follows:

(i) *Channel Symmetricity*: For the symmetric channel, the cognitive radio transmitter (CR-Tx) and cognitive radio receiver (CR-Rx) have the same available channels. The available channels indicate the number of vacant channels for rendezvous. In other words, the number of empty (and common) channels is the same as the total number of channels. The number of empty channels, on the other hand, could be smaller than the actual number of channels. In addition, the number of common channels might be smaller than the number of empty channels. If the number of empty or common channels is less than the total number of channels, the case is classified as the asymmetric channel.
(ii) *Channel Synchronicity*: There is a lack of time alignment for rendezvous initiation because every CRU node may not have the same time slot to start the rendezvous process. This is known as the asynchronous channel state. The synchronous condition indicates that CR-Tx and CR-Rx have precisely the same rendezvous activation time.
(iii) *Anonymous Condition*: The anonymous situation states that any CRU node is unaware of another node’s strategies. Some possible strategies are the channel selection process, implemented algorithm and channel vacancy condition, node information such as node id, wake-up time, etc.
(iv) *Channel Homogeneity*: When both channel number homogeneity (symmetric condition) and time homogeneity (synchronous status) prevail for the given nodes, it is referred to as a homogeneous (fully homogeneous) channel condition. Apart from that, a partially homogeneous state is predicted, in which channel homogeneous channel number exists but time synchronisation does not.
(v) *Channel Heterogeneity*: Heterogeneous channels are those that have a wide variety of typical vacant channels for various nodes. In other words, it lacks both channel symmetry and synchronicity.
(vi) *Deterministic Condition*: The predefined rule of channel hopping or visiting channel for CR-Tx and CR-Rx are set such that, both ends are exactly aware of its counterpart. For instance, at any time slot, CR-Tx (resp. CR-Rx) knows the channel selected by CR-Rx (resp. CR-Rx). In the partially deterministic approach, each node only has limited knowledge of other nodes’ strategies and channel conditions.
(vii) *Non-deterministic Condition*: Non-deterministic rendezvous, on the other hand, refers to a case in which no one recognizes the other nodes’ rendezvous strategy or methodology or channel selection process.

The non-assisted rendezvous is divided into two categories based on the prediction of the status of other nodes—deterministic and non-deterministic. The deterministic (and/or partially deterministic) conditions are dependent on a combination of channel synchronicity, symmetry, and known conditions of each other node’s channel selection strategies. Non-deterministic, on the other hand, has the opposite attributes. As a result, in comparison to the latter conditions, the former case simplifies the rendezvous mechanism using the concepts of simplified assumptions. Blind rendezvous, without a centralized controller, has therefore gained tremendous interest for the practical network scenarios. It is necessary to explore appropriate blind rendezvous (non-deterministic channel) conditions to improve rendezvous performance in terms of time and energy cost. Many recent papers on rendezvous presume two conditions of the channel—symmetric and asymmetric. In the literature below [14,15], rendezvous for homogeneous conditions such as symmetric channels is noted. Rendezvous techniques based on the union of disjoint differential sets were proposed in [14] and performance was shown in average time and efficiency. A prime number-based rendezvous scheme was studied in [15] for a semi-heterogeneous network such as the symmetric asynchronous channel. For the above semi-heterogeneous condition, the peak rendezvous times were superior to those achieved with conventional methods. Prime number theory-based rendezvous has also achieved excellent results for blind rendezvous under non-deterministic arrangements. Some mentionable recent activity on unassisted as well as heterogeneous rendezvous with a single radio includes [16,17,18,19,20,21]. With the modular clock-based prime number theory [16], Nick et al. proposed supported rendezvous for CR. However, because of unpredictable random sequences, the utilization of the prime number theory is limited. The articles [17,18,19] carried out a statistical distribution analysis for efficient and guaranteed rendezvous. In a heterogeneous scenario, the rendezvous algorithm with the Chinese remainder theorem for the multi-radio method was proposed by [20]. To achieve rendezvous in a limited period of time, Shin et al. proposed a deterministic channel rendezvous method [21]. Rendezvous based on prime number theory had outstanding results for blind rendezvous with non-deterministic arrangements, which were evident in [22]. Jump-stay rendezvous with deterministic progression was suggested for both the symmetric and the asymmetric channels in [22]. In addition, prime number theory was changed from its original sequences to make semi-random sequences [23]. A fast and blind rendezvous scheme considering jamming attack was proposed by [24]. A robust rendezvous with less time to rendezvous (TTR) and MTTR was shown, considering the asynchronous channel scenario. Rendezvous based on primitive roots was proposed for different channel conditions such as symmetric and asymmetric in [25] and the achievement of a common channel within a limited time cycle was promising. However, with the uncertainty of channel symmetry and time synchronization at either end of CR nodes, a universal algorithm is needed for further improvement. A deterministic succession-based rendezvous for a better outcome was analysed in [26] and they proposed a channel rendezvous with maximum diversity. Bian et al. suggested an asynchronous channel-hopping scheme [27]. A 48-bit node identification (ID) was shown in the proposed scheme [27], but the presented TTR was below the effectiveness of the assumption of ID. The entirety of the above work relies on the technique of single-radio-based strategies.

Rendezvous techniques for CR devices with multiple radios have been suggested in the following articles. In the multi-radio concept, more than one radio is mounted on any device, and rendezvous is attempted with parallel channel-hopping for any device. A channel-hopping scheme for multi-radio wireless networks was suggested in [28]. A reduction of time for multi-radio-based rendezvous was required to explore for further improvements of rendezvous. The article [29] demonstrated successful rendezvous with the pair-wise assumption in a multi-hop environment. The presumption, on the other hand, has very little practical rendezvous application for a blind rendezvous network without any known control channel. For enhanced MTTR, multi-radio-based rendezvous for the homogeneous condition was recommended in [30]. Multiple-radio-based rendezvous with upper bound was illustrated in [31]. The average time for rendezvous was reduced significantly with different combinations of radios. Efficient rendezvous based on the matrix was proposed in [32], where the process was based on a local channel set. Different scenarios were suggested in [33,34,35], where authors achieved quick rendezvous with the presence of multiple radios for different heterogeneous conditions. The contributions of the most notable recent article [34] having blind and heterogeneous environment are as follows, (i) it proposed blind rendezvous strategies in the presence of distributed heterogeneous cognitive radio network (CRN); (ii) it achieved quick rendezvous with concepts such as single-radio, multi-radio and hybrid radio; (iii) for effective rendezvous, distinct rendezvous strategies for different combinations of radios were presented; (iv) the authors calculated TTR’s potential upper limit as well as the optimum number of radios. However, the following deficiencies for [34] must be investigated for further development: (i) the need for more closely bound rendezvous is inevitable; (ii) the efficiency of radio allocation was dependent on the jump-radio and stay-radio of the CR-Tx and CR-Rx, and the performance is unstable due to the lack of radio selection independence at either end. (iii) there were no convincing reductions in TTR for single and multiple radio combinations, necessitating further analysis; (iv) MTTR was calculated using a heuristic approach; however, probability-based rendezvous accuracy estimation is more consistent; (v) the MTTR for all scenarios must be reduced significantly for the smooth rendezvous.

For all aforementioned multi-radio based rendezvous techniques, the improvement of TTR was clarified considering different scenarios (both homogeneous and heterogeneous). From their presented simulation outcomes, it is observed that deterministic and semi-random sequences show promising rendezvous results for homogeneous and semi-homogeneous conditions. However, in heterogeneous instances, for the random variations of scenarios, efficient compatibility of deterministic and semi-random sequences is not noticeable. In addition, heterogeneous algorithms incur complexity when applied to homogeneous and semi-homogeneous radio environments. Moreover, very few works present a methodology that fits well in anonymous, heterogeneous and asynchronous conditions. Further, most papers employ traditional heuristic rendezvous proofs for specific cases without providing probabilistic guarantees. Again, for single or multiple radios, no closed-form solutions for random sequence approaches such as modular prime number methods are available.

In this paper, we have proposed a multi-radio-based rendezvous with the prime number modular method for blind/anonymous and fully heterogeneous conditions. As the prime number modular technique creates a random sequence, it can be best applicable to the heterogeneous scenario. We derive the statistical distribution of rendezvous time based on the given channel cycle for the prime number modular-based random sequence. Further, a theoretical framework for rendezvous probability with a closed-form expression for guaranteed rendezvous is established. The required time and the optimum numbers of radios for guaranteed rendezvous are determined on the basis of total probability. A very close match between simulation and theory is observed through Matlab simulation, and the maximum rendezvous time is compared with the recently published result of blind and heterogeneous rendezvous schemes [34]. To the best of the authors’ knowledge, there are no other literature results that provide the statistical distribution, or a closed-form expression, for guaranteed rendezvous under the scenario presented here.

## 2. System Model

### 2.1. Network Model

**Figure 1:**
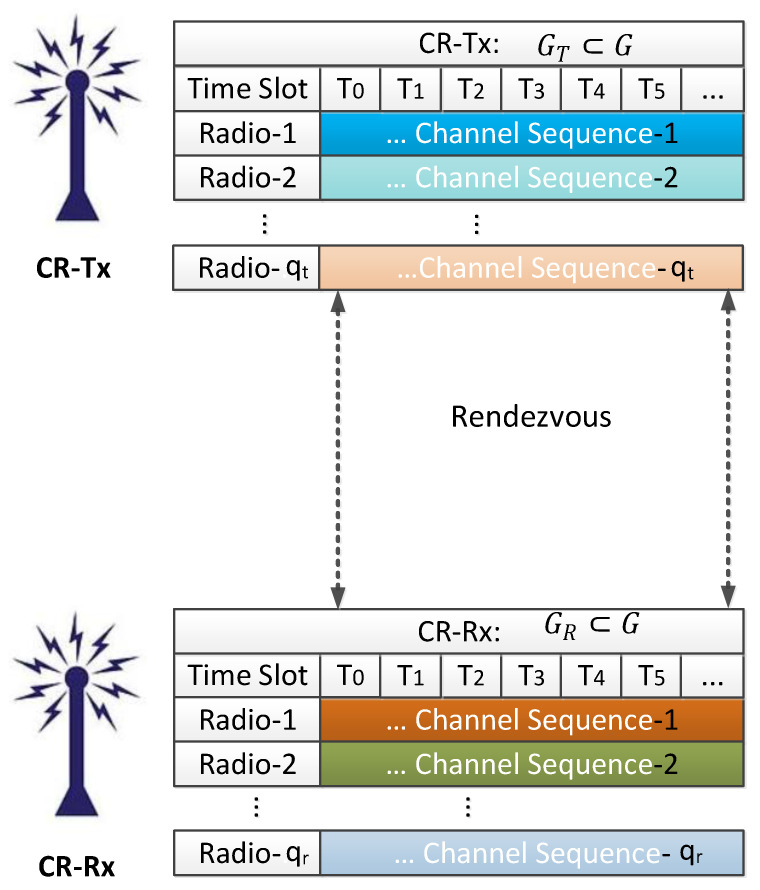
Block Diagram of Time and Channel Sequence for Multi-Radio Based Rendezvous.

**Figure 2:**
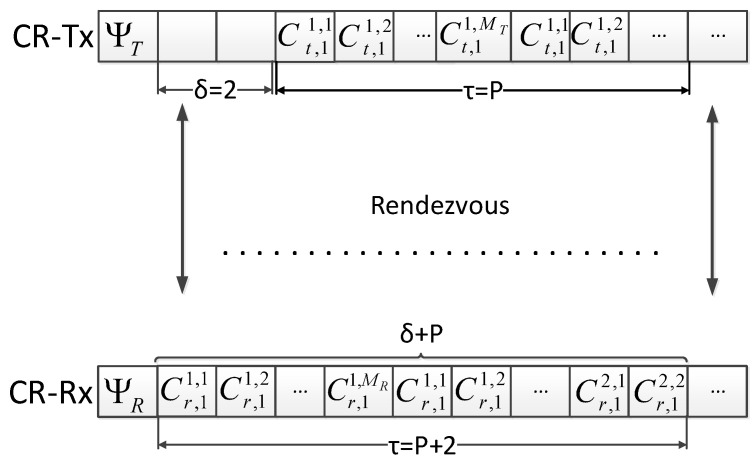
An example scenario of time delay for δ=2 with single sequence.

In this paper, we study a distributed CRN consisting of a CR-Tx and a CR-Rx seeking to communicate with each other on a common frequency channel. We assume a decentralized system with *M* channels, where neither CR-Tx nor CR-Rx has any knowledge of the vacant channel availability. Both secondary users (SUs) are aware of the available *M* channels, but the CR-Rx is not aware of the channel chosen for transmission by the CR-Tx as it is a decentralized network with a lack of signalling channels. (In this article, cognitive radio user (CRU) and secondary user (SU) are used synonymously.) The set of available channels is given by $G=\{C^{1},C^{2},\ldots ,C^{M}\}$. CR-Tx and CR-Rx channels are $G_{T}\subset G$ and $G_{R}\subset G$ respectively. The number of CR-Tx and CR-Rx channels are $M_{T}=\left|G_{T}\right|$ and $M_{R}=\left|G_{R}\right|$, respectively. The available vacant channels that are common to both ends are $\bar{G}=G_{T}\cap G_{R}$ and $\bar{M}=\left|\bar{G}\right|$. In addition, assuming the start and end times are not synchronized for both the SUs, we consider imperfect channel synchronization. As no synchronization of channels is available, no clock alignment of starting channels between CR-Tx and CR-Rx exists. Each radio attempts to achieve rendezvous with multiple radios mounted on it (as shown in the block diagram of Figure 1). A random rendezvous initiation delay (δ) is considered in the range (expressed in number of slots) $\left[0,min\left(M_{T},M_{R}\right)\right]$ as shown in Figure 2. Using the prime number technique presented below, the two SUs intelligently achieve consensus on the transmission channel.

### 2.2. Prime Number Theory for Rendezvous Process

The theory of prime numbers [16,20] is applicable for rendezvous, with channel arrangement based on two factors: (a) starting with a channel, called the initial channel selection, and (b) choosing the channels with fixed intervals, called the jumping rate. We assume that the CR-Tx and CR-Rx execute the modular clock algorithm with jumping rates of α,β respectively. Any initial channel selection is performed for each j’th channel for the n’th time slot. Thus, the channels for CR-Tx are:

(1) $$C_{t}^{j}\left[n+1\right]=mod\left\{C_{t}^{j}\left[n\right]+\alpha ;P\right\}$$

and CR-Rx creates channels as follows:

(2) $$C_{r}^{j}\left[n+1\right]=mod\left\{C_{r}^{j}\left[n\right]+\beta ;P\right\},$$

where (1≤α<P,1≤β<P. *P* can be written as,

(3) $$P=arg\;\underset{P^{\prime }}{min}\left\{P^{\prime }\geq M\right\},$$

where $P^{\prime }$ is the prime number, and mod indicates modulus operation. According to the prime number modular method [16], rendezvous occurs within *P* time slot if two prerequisites are satisfied: (i) any channel choice for both CR-Tx and CR-Rx with $P=M=M_{T}=M_{R}$ have channels with the same order and (ii) the jumping rate for CR-Tx and CR-Rx must be different, that is, α≠β. In this work, because we have the blind rendezvous condition, there is no assurance of the two conditions above. Thus, we assume $P\neq M\geq \;\left(M_{R},M_{T}\right)$ along with the presence of delay in the system, which points to one of the key novelties and contributions presented in this paper. The proposed strategy for cycle and sequence generation is explained in the next sub-sections.

## 3. Proposed Rendezvous Method Based on Prime Number Theory for Multi-Radio System

The channel cycle and sequence generation are shown in this section. The basic channel formation with prime number theory is described below (Section 3.1), and the extension of channel formation with the multiple radios is explained in Section 3.2, following the same theory. The block diagram of time slot and channel sequence for a multiple-radio scenario is displayed in Figure 1.

### 3.1. Prime Number Theory Based Channel Cycle

In our proposed system model of anonymous channel scenarios, the available *M* channels are known to both the CRUs. However, the CR-Tx is not aware of the common and selected channel by the CR-Rx for transmissions. The channel cycle and sequence generation are performed in two steps. First, a basic channel cycle of *P* length based on *M* channels is created. The available $M_{T}$ $\left(resp.\;M_{R}\right)$ channels are kept in their respective time position (with ascending order). Because heterogeneous channels prevail, all *M* channels are not vacant. Therefore, occupied channel positions are filled from the available channels with a total of *M* vacant channels. The channel allocation method is performed in such a way that no channels are chosen more than once for a limited number of occupied positions. *P* is the minimum prime number greater than or equal to *M*, which is defined by Equation (3). Then, for the channel case of P>M, random channel selection of each cycle is applied for PM positions, following the uniform probability of each selected channel. If the (P−M)<M condition occurs, the uniform probability of selection is implied such that no current channel is chosen twice for P−M slots. Because channel asymmetry is available, the prevailing vacant channels have randomness. Therefore, there is no certainty that all selected channels are vacant common channels. The random channel selection ensures a uniform rate of channel selection from unknown channels. Second, from our formed P channels, each channel sequence consisting of multiple cycles is created with prime number theory. When each arbitrary cycle is created in any sequence, a random jumping rate and initial channel are chosen maintaining dissimilarity between two cycles. The channel cycles in any sequence of each CRU are repeated with the aforementioned methods to achieve rendezvous between CR-Tx and CR-Rx. Further, we define the maximum number of repetitions for the cycle as σ, and if the maximum repetition (σ) is reached in any sequence without achieving rendezvous, the radio pairs then abandon the rendezvous. The channel sequences with multiple radios are shown in the sub-section below.

### 3.2. Multi-Radio-Based Channel Sequence

We now extend the network model to multi-radio-based transmitter and receiver systems. The key objective here is to achieve rendezvous by hopping on the parallel sequence for each radio, and hence utilizing the spatial diversity for accomplishing rendezvous faster than for a single-radio system. In doing so, we assume that the multi-radio system is multiple input multiple output (MIMO) capable with space-time, number of radios at CR-Tx and CR-Rx, respectively. For each radio, a channel sequence is generated that consists of several independent cycles of P length. Each cycle is formed on the basis of a random initial channel and different jumping rates for each radio. This means that every sequence from $q_{t}$ radios at the transmitter will be used to perform rendezvous by every sequence from the $q_{r}$ radios at the receiver, which gives us $Q=q_{t}q_{r}$ independent sequences of opportunities to achieve rendezvous as oppose to a single cycle opportunity with a single radio $\left(q_{t}=q_{r}=1\right)$ system. The channel sequence of the s’th radio for CR-Tx and CR-Rx is symbolized by $\Psi_{T,s}$ and $\Psi_{R,s}$ respectively, and can be written as follows:

(4) $$\begin{array}{c} \Psi_{T,s}=\left\{\underset{P}{\underset{︸}{C_{t,s}^{1,1},C_{t,s}^{1,2},\ldots ,C_{t,s}^{1,M_{T}},C_{t,s}^{1,1},\ldots }}\underset{P}{\underset{︸}{C_{t,s}^{2,1},C_{t,s}^{2,2},\ldots ,}}\ldots \right\} \end{array}$$

(5) $$\begin{array}{c} \Psi_{R,s}=\left\{\underset{P}{\underset{︸}{C_{r,s}^{1,1},C_{r,s}^{1,2},\ldots ,C_{r,s}^{1,M_{R}},C_{r,s}^{1,1},\ldots }}\underset{P}{\underset{︸}{C_{r,s}^{2,1},C_{r,s}^{2,2},\ldots ,}}\ldots \right\}, \end{array}$$

where in $C_{t,s}^{i,j}$ and $C_{r,s}^{i,j}$, *j* is the channel number with 1≤j≤P, and *i* is the cycle number with 1≤i≤σ. In a multi-radio system having more than one radio, all CR-Tx parallel sequences $\left(\Psi_{T,q_{t}}\right)$ listen to all similar sequences of CR-Rx $\left(\Psi_{R,q_{r}}\right)$ for rendezvous. For any sequence of CR-Tx and CR-Rx, rendezvous is achieved during time slot *n* when both ends use the same channel to transmit and receive, that is, when $C_{t,s}^{i,j}\left[n\right]=C_{r,s}^{i,j}\left[n\right]$. Thus, the TTR (τ∈N) is the corresponding time from start to end for finding a common channel (that is, achieving rendezvous). We define the repetition rate for channel $C^{j}$ within the cycle of *P* time slots as $\rho_{j}$ given by:

(6) $$P=\rho_{j}\max\left(M_{T},M_{R}\right)+\epsilon ,$$

noting that $\rho_{j}$ is an integer value as per the definition, and for every channel *j* and every *P* cycle, $\rho_{j}$ is the same; therefore we define $\rho_{j}=\rho$ for all j. In (6), ϵ is some value less than $\max(M_{T},M_{R})$. We also define the number of uncommon channels (μ) in one single cycle with *P* time slots as:

(7) $$\mu =P-\rho *\bar{M}.$$

As the number of unknown channels increases in a given cycle, there is indeed a greater likelihood that rendezvous will not occur. For no delay and with a single radio, CR-Tx and CR-Rx are able to search the channel in a single cycle and rendezvous can be achieved within the first cycle of P time slots. However, the TTR with the cost of δ>0 requires more than one cycle of *P* time slots.

An example scenario of rendezvous delay and required time for the simple case of a single radio is shown in Figure 2 for δ=2. Rendezvous is not achieved in the first cycle of P time slots, and therefore, additional time is required for rendezvous. Thus, the total number of channel cycles is γ=σQ.

## 4. Rendezvous Probability and Optimal Parameters for the Proposed Method

### 4.1. Rendezvous Probability

In this section, we present a probabilistic analysis of the proposed method to find the rendezvous probability and the MTTR. We define two probabilities: the rendezvous probability for a single cycle as Pr[τ≤P], and the probability of rendezvous for all σ cycles as Pr[τ≤Pσ]. The rendezvous probability for any independent (single) cycle with a sequence of δ=0 and is given for a single radio as follows:

(8) $$Pr\left(\tau \leq P\right)=\frac{P!-D_{P}}{P!}.$$

$D_{P}$ is the number of derangements. The derivation of (8) is given in Appendix A. Because the rendezvous per every cycle is independent, the total rendezvous probability ($p_{{}_{R}}$) for δ=0 is given by:

(9) $$p_{{}_{R}}=\sum_{q=1}^{Q}Pr\left(\tau \leq P\sigma \right).$$

The derivation for $p_{{}_{R}}$ is provided in Appendix A. In order to derive this probability, let us define the event $e_{c}$ as the event where rendezvous is achieved in any of the σ cycles in any of the *Q* radios, where 1≤c≤γ. The maximum number of cycles for *Q* radios is given by γ=Qσ. Therefore, Equation (9) becomes:

(10) $$p_{{}_{R}}=\sum_{q=1}^{Q}Pr\left(\tau \leq P\sigma \right)=Pr\left(\overset{\gamma }{\underset{c=1}{⋃}}e_{c}\right).$$

Using the inclusion-exclusion principle [36], the derivation is illustrated in the below part.

Total Rendezvous Probability ($P_{R}$) Derivation:

The Equation (10) can be written from inclusion and exclusion principle [36] as below:

(11) $$\begin{array}{cc} Pr(\overset{\gamma }{\underset{c=1}{⋃}}e_{c})=\; & \sum_{c=1}^{\gamma }Pr\left(e_{c}\right)-\sum_{1\leq c<d\leq f}^{}Pr\left(e_{c}\cap \tau_{d}\right)+\sum_{1\leq c<d<f\leq \gamma }^{}Pr\left(e_{c}\cap \tau_{d}\cap \tau_{f}\right)-\ldots \\ \; & +{\left(-1\right)}^{\gamma -1}\sum_{1\leq c<d\ldots <\gamma -1\leq \gamma }^{}Pr\left(e_{c}\cap e_{d}\cap \ldots e_{\gamma }\right)\\ \; & =\sum_{S\subset [\gamma ],S\neq \emptyset }^{}{\left(-1\right)}^{|S|+1}Pr\left(\underset{c\in S}{⋂}e_{c}\right). \end{array}$$

The probability of independent event is expressed as:

(12) $$Pr\left(e_{c}\cap e_{d}\cap e_{f}\cap \ldots .\right)=Pr\left(e_{c}\right)*Pr\left(e_{d}\right)*Pr\left(e_{f}\right)*,\ldots$$

For any *r* number out of γ, the result is obtained from the combination: ∑1r.=γr. Moreover, since every cycle has the same length, the equal channel is available in each channel cycle. As the rendezvous probability for any random event is the same such as, $Pr\left(e_{c}\right)=Pr\left(e_{d}\right)=Pr\left(e_{f}\right)$ and so on, the rendezvous for any single sequence is denoted by simplified form $Pr(e_{1})$. For instance, with $k_{1}$ number of events above equation can be written as:

(13) $$Pr\left(e_{1}\cap e_{2}\cap \ldots .\cap e_{k_{1}}\right)=Pr{\left(e_{1}\right)}^{k_{1}}.$$

Therefore Equation (11) can be written as:

(14) Pr(⋃c=1γec)=γ1∗Pr(e1)1−γ2∗(Pr(e1)2+γ3∗Pr(e1)3−…..+(−1)γ−1γγ∗Pr(e1γ=∑c=1γ(−1)c−1γc(Pr(e1))c.

The probability for any single event, that is, any single cycle is given in above Equation (A8). The Equation (14) is written by following Equation (A8) as below:

(15) Pr(⋃c=1γec)=∑c=1γ(−1)c−1γcP!−DPP!c.

We can rewrite (10) as:

(16) $$Pr\left(\overset{\gamma }{\underset{c=1}{⋃}}e_{c}\right)=\sum_{S\subset [\gamma ],S\neq \emptyset }^{}{\left(-1\right)}^{|S|+1}Pr\left(\underset{c\in S}{⋂}e_{c}\right).$$

Because the rendezvous for every cycle is independent, $Pr\left(⋂e_{c}\right)=Pr{\left(\tau \leq P\right)}^{c}$. The probability of rendezvous for any single event Pr(τ≤P) is obtained from (8). Therefore, from (8) above (16) can be represented by:

(17) pR=∑c=1γ(−1)c−1γcP!−DPP!c.

### 4.2. Optimized Parameters for Rendezvous

For our target rendezvous probability ($\hat{p_{{}_{R}}}$) with given γ, the minimum number of radios ($Q_{min}$) and the minimum required channel cycle ($\sigma_{min}$) are represented by (18) and (19), respectively, as follows:

(18) $$Q_{min}=arg\min_{Q}\left[p_{{}_{R}}\geq \hat{p_{{}_{R}}}\right]$$

(19) $$\sigma_{min}=arg\min_{\sigma }\left[p_{{}_{R}}\geq \hat{p_{{}_{R}}}\right].$$

Based on the system design, MTTR is measured on the maximum number of cycles with delay (δ>0):

(20) $$MTTR=P\sigma +\delta ;\;for\;p_{{}_{R}}\geq \hat{p_{{}_{R}}}.$$

## 5. Simulation Result

In this part, we’ve summarised the findings of our proposed scheme with the analytical perspective. The statistical characteristics of rendezvous performance are calculated using Monte Carlo simulations. For the measurement of MTTR and optimal resources based on total probability, we performed the simulations using Matlab. We choose [34] as a standard to which we may compare our findings. This recently published paper has a better overall outcome in minimizing MTTR with blind and heterogeneous channel conditions. Despite the fact that the above reference paper considered three conditions—single, hybrid, and multiple radios, we compared our results for the latter two related schemes (hybrid and multiple) with our idea. Our proposed concept for rendezvous takes into account blind and heterogeneous conditions with multiple radios, which is the perfect fit for the said paper to compare. In the presented simulation, the data are measured for rendezvous between a transmitter (CR-Tx) and a receiver (CR-Rx). Under the asymmetric channel availability model in which SUs have various asynchronous conditions, the systems are simulated. To determine rendezvous, however, there is at least one available channel between CR-Tx and CR-Rx. The simulation parameters are demonstrated in Table 1. To validate the simulation in Matlab, between $10^{5}$–$10^{6}$ runs are used for each case measurement. For different criteria, the outcomes are justified for a minimum of 10 to 150 channels, where the number of vacant channels for transmitter ($M_{T}$) and receiver ($M_{R}$) is less than the total channels. Moreover, the number of common channels is less than or equal to CR-Tx or CR-Rx channels. A random rendezvous activation delay is considered that can vary from nil to a maximum value of $min(M_{T},M_{R})$. The target probability for successful rendezvous is set to a minimum of 0.99, and justification is presented with above 0.99 and 0.9999 for further clarification. The outcomes are shown for different numbers of radios ($q_{t},q_{r}$), where a minimum of 1 to maximum 4 radios are utilized. The number of radios at either CR-Tx or CR-Rx may not be necessarily the same.

The number of required channel cycle varies based on the number of radios and at least one channel cycle (σ≥1) is required for achieving a successful rendezvous operation.

**Figure 3:**
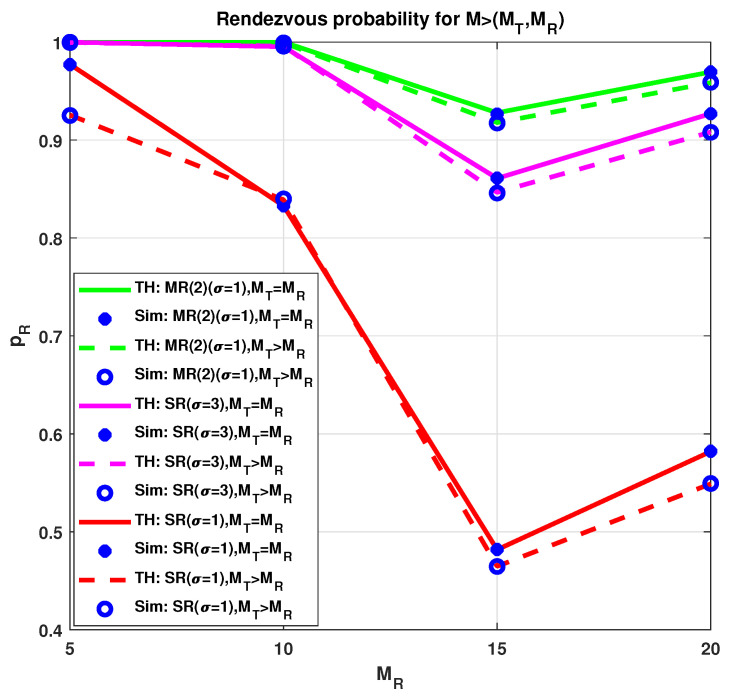
Rendezvous probability with M=25 channels.

In Figure 3, rendezvous probability for two scenarios of the channel with conditions $M>(M_{T}=M_{R})$ and $M>(M_{T}=M_{R}+5)$ are shown for a common channel $\bar{M}=M_{R}$. The total number of channels is M=25. In general, the letters SR stand for single radio, MR(qt=qr) for multi-radio, and performance is based on an equivalent number of radios (qt=qr). Along with the different number of radios, the number of channel cycles is varied for showing varying performances. The growth in the probability of rendezvous for a single radio is noticeable when the number of channel sequence lengths is higher, and opposite trend is evident for less sequence length. For instance, with $q_{t}=q_{r}=1$ radios and σ>1 length, the probability of rendezvous is higher than for σ=1 length. In addition, for multi-radio assisted rendezvous higher rendezvous probability is evident. Furthermore, for $\bar{M}=M_{T}=M_{R}$ case, the rendezvous probability is higher than that of $\bar{M}<\left(M_{T},M_{R}\right)$. Searching space for the latter case is increased for the higher number of uncommon channels, thus more time is required for achieving rendezvous.

**Figure 4:**
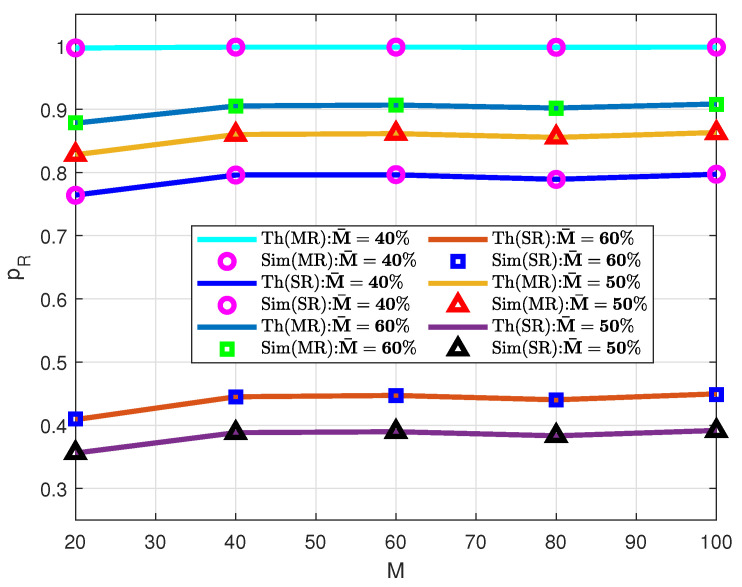
Rendezvous probability for different number of channels.

The total rendezvous probability for the different number of channels is demonstrated in Figure 4 with single and multiple radios. It is necessary to mention that for the multiple-radio case, two radios are assumed on each side. For the results, the number of channel cycles is fixed to one. Different fixed percentages of the number of common channels reveal the variation of results. The vacant channels for CR-Tx and CR-Rx are 10% higher than common channels, that is, $M_{T}=M_{R}=\bar{M}+M*10\%$. In other words, 10% unknown channels are available on both transmitter and receiver sides.

From the data (Figure 4), it is evident that the least number of common channel is 40% and this has the higher value of rendezvous probability. As a channel repetition rate of two (ρ=2) is ensured within each channel cycle, the rendezvous probability is obviously higher than that of a single repetition rate. For the remaining two common channels ($\bar{M}=50\%$ and $\bar{M}=60\%$), the channel repetition rate is single within the channel cycle. Therefore, a higher rendezvous probability is observed for 60% of common channels. Additionally, rendezvous with multiple radios has a higher probability compared with a single radio. For instance, with $\bar{M}=40\%$ channels, the rendezvous probability is above 0.99 with every number of total channels. However, probability varies from 0.77 to 0.81 with the results of single-radio rendezvous.

**Figure 5:**
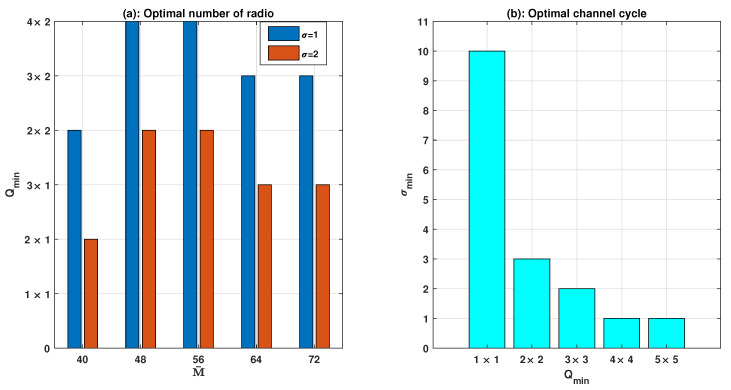
Optimal number of radios and channel cycles.

The necessary number of radios and channel cycles for target rendezvous probability ($p_{{}_{R}}>0.99$) are displayed in Figure 5a,b, respectively. The minimum number of radios for both sides is measured by $Q_{min}=min\left(q_{t}\times q_{r}\right)$ or vice versa for $q_{t}$ and $q_{r}$; and we have verified Figure 5a for the following radio combinations, $Q_{min}=1\times 1,\ldots ,4\times 2$ with M=80. The channel sequences with P length require a greater number of radios for achieving expected rendezvous, and an opposite trend is observable for σ=2 i.e., 2P sequence length. For 50% common channels, the minimum number of radios is required to fulfil target rendezvous probability. A sharp increase in the number of radios is required for 48 channels or more, and a decreasing trend is seen for the highest number of channels (72).

For two-channel situations, Figure 5b shows rendezvous with common channel $\bar{M}=50$. There is an equal number of radios for CR-Tx and CR-Rx for the considered scenario. For an increase in the number of radios, fewer channel cycles are needed for guaranteed rendezvous (satisfying $\hat{p_{{}_{R}}}$). For illustration, a single radio requires a minimum of 10 channel cycles for achieving rendezvous. On the other hand, for 4×4 and 5×5 scenario, a single channel cycle is sufficient for expected rendezvous probability. Thus, 4×4 radios are optimal for the rendezvous with the given number of channels.

**Figure 6:**
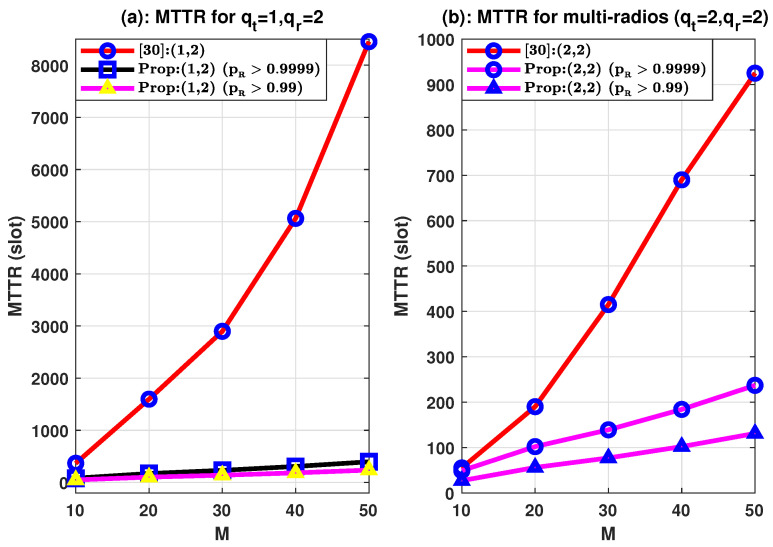
MTTR for different channels (*M*) with $\bar{M}=0.5M$.

The MTTR is displayed in Figure 6 based on the target rendezvous probability $\hat{p_{{}_{R}}}$ for the heterogeneous case $M_{T}=M_{R}=0.7M\left(>\bar{M}\right)$ having the common channel $\bar{M}=0.5M$. The paper [34] considers heterogeneous conditions with a lower number of common channels as compared with available channels for CR-Tx and CR-Rx and randomly varies 0.2M channels which are uncommon. There is a delay with $\delta =min(M_{T},M_{R})$. To justify with higher accuracy, the required MTTRs for guaranteed rendezvous ($p_{{}_{R}}>0.99$) and ($p_{{}_{R}}>0.9999$) are compared with the said paper.

The MTTR for the aforementioned article has a higher rendezvous time in all cases compared with our proposed system. For example, for $p_{R}>0.9999$ and $p_{R}>0.99$ MTTRs, our proposed scheme (Figure 6a) of total 10 channels is reduced to 22.28 percent and 13.3 percent, respectively. The largest reductions to 5 and 3 percent respectively are found for overall 50 channels (for the above two probabilities). Furthermore, for the proposed case of multiple radios (Figure 6b), MTTRs are reduced to 89 and 51 percent for $p_{R}>0.9999$ and $p_{R}>0.99$, respectively, with total 10 channels. The reductions are 25.60 percent and 14 percent, respectively, for the total 50 channels (for the above two probabilities). Therefore, it can be concluded that the higher decrease (gain) of MTTR occurs when the number of channels is higher. Furthermore, as shown in Figure 6, a combination of single and multi-radio has the highest rendezvous time in paper [34]. It is also found that our proposed approach achieves considerably smaller MTTR for guaranteed rendezvous with multiple radios at the sender and receiver ends. Thus, any combination of radios can be adapted to our proposed system, unlike other approaches. Therefore, as opposed to the algorithm designed with modified jumping and staying sequence by the conventional method, our proposed prime number-focused rendezvous outperforms for the blind and fully heterogeneous contexts.

## 6. Conclusions

In this article, we present a multi-radio rendezvous algorithm for non-assisted rendezvous conditions that use prime number methods. For the most efficient results, blind/anonymous and fully asynchronous scenarios are considered. For a variety of scenarios, the statistical distribution of guaranteed rendezvous in terms of rendezvous probability is explored. A theoretical derivation of the probability of rendezvous for various time sequences and required maximum rendezvous time is also established for blind rendezvous. The proposed approach reveals time reductions in terms of MTTR for a variety of channel conditions as well as the number of radios. The theoretical and simulation results are seen to be remarkably similar, indicating that the proposed approach performs as anticipated. The findings of our presented idea are compared to a recently published blind and heterogeneous rendezvous focused on multiple radios. For our proposed solution, the comparative findings show a significant time gain with guaranteed rendezvous.

## Figures and Tables

**Table 1:** Simulation parameters.

Parameter	Value
Number of Monte Carlo runs	$10^{5}$–$10^{6}$
Number of total channels (M)	10–150
Number of CR-Tx and CR-Rx channels $\left(M_{T},M_{R}\right)$	<M
Number of common channels $\left(\bar{M}\right)$	<M
Delay (rendezvous activation)	$0\;to\;min(M_{T},M_{R})$
Number of radios $\left(q_{t},q_{r}\right)$	$1\;\leq q_{t},q_{r}\leq 4$
Target rendezvous probability ( ${\hat{p}}_{R}$ )	${\hat{p}}_{R}\geq 0.99$
Number of channel cycle (σ)	≥1

## Data Availability

Not applicable.

## Appendix A. Derivation of Rendezvous Probability for Single Cycle

The probability of rendezvous is calculated from the well-known theorem: derangement [37]. For the given set, [P]={1,2,3,…,P}, ϕ is the permutation of *P*. A derangement is the permutation for ϕ(y)≠y for all y∈P. The total rendezvous probability is measured from total permutation for any given number of objects and the number of derangement for that objects. Let $D_{P}$ be the number of derangements for P objects, with the assumption that all objects are available and distinct. Then $D_{P}$ becomes:

(A1) $$D_{P}=P!\sum_{l=0}^{P}\frac{-1^{l}}{l!}$$

However, for the heterogeneous conditions of our system model, each channel is not available with full certainty for any given cycle. Moreover, each channel may have more than a single repetition rate (ρ>1). Therefore, if any unknown channel is available or any known channel is repeated more than once, the derangement with the above equation are difficult to articulate. Thus, the number of derangement is calculated from the rook theory [38] with the association of Laguerre polynomial [39,40]. A famous formula from rook theory [38], proven using inclusion-exclusion is used for the number of derangement for the given objects. If *B* is a “board”, a subset of the n×n grid [n×n], then let $r_{k}$ be ways of placing *k* elements on the board *B* with no two in the same row or column (i.e., the number of ways of placing *k* rooks from chess that cannot attack each other. rook polynomial $r_{B}\left(x\right)$ of a board B⊆[n×n] to be as follows:

(A2) $$r_{B}\left(x\right)=\sum_{k=0}^{n}r_{B,k}*x^{k}$$

which is the rook polynomial for B. if $B_{1}\subseteq \left[n_{1}\times n_{1}\right],B_{2}\subseteq \left[n_{2}\times n_{2}\right]$ and so on, then equation becomes:

(A3) $$r_{B}\left(x\right)=r_{B_{1}}\left(x\right)*r_{B_{2}}\left(x\right)*\ldots ..$$

The $r_{B}\left(x\right)$ is illustrated with Laguerre polynomial in reference [38] and any of $r_{B}{}_{j}\left(x\right)=L{}_{\rho_{j}\left(x\right)}$ is written in the below Equation (A4). Consider the channel cycle composed of channel-1, channel-2, ..., channel-$\bar{M}$ with ρ≥1. For any channel replication ρ≥1, the Laguerre polynomial can be written as:

(A4) Lρj(x)=∑k=0ρj(−1)k∗ρjk∗k!∗xρj−k

where $\rho_{j}\in \rho$. For the unknown channels, putting unknown objects i.e., $\rho_{j}=0$, laguerre polynomial can be expressed as [40]:

(A5) $$L_{\rho_{j}\left(x\right)}^{\prime }=x^{k}$$

For all given objects, above two equations are as follows:

(A6) $$d=\prod_{j=1}^{\bar{M}}L_{\rho_{j}\left(x\right)}*\prod_{j=\bar{M}+1}^{\mu_{1}+\bar{M}}L_{\rho_{j}\left(x\right)}^{\prime }$$

Let θ to be the linear function on polynomials mapping [3] $x^{\rho_{j}}$ to $\rho_{j}!$, number of derangements $D_{P}$ is calculated as:

(A7) $$D_{P}=\theta \left(d\right)$$

The probability of rendezvous for any channel cycle is indicated by $Pr\left(\tau \leq P\right)$ and given as:

(A8) $$Pr(\tau \leq P)=\frac{P!-D_{P}}{P!}$$

P! is the *P* number of permutation.
